# Supplementary material for: Multidisciplinary Collaboration for the Optimization of Antibiotic Prescription: Analysis of Clinical Cases of Pneumonia between Emergency, Internal Medicine, and Pharmacy Services
Source: Antibiotics (Basel). 2022 Sep 30;11(10):1336. doi: 10.3390/antibiotics11101336 (PMC9598292; doi:10.3390/antibiotics11101336)
Supplement: Supplementary file 1 [file antibiotics-11-01336-s001.zip › antibiotics-1863856-supplementary.pdf]

Figure S1. Antibiotic consumption: DDD/1000 visits in emergency department in 2019-2020

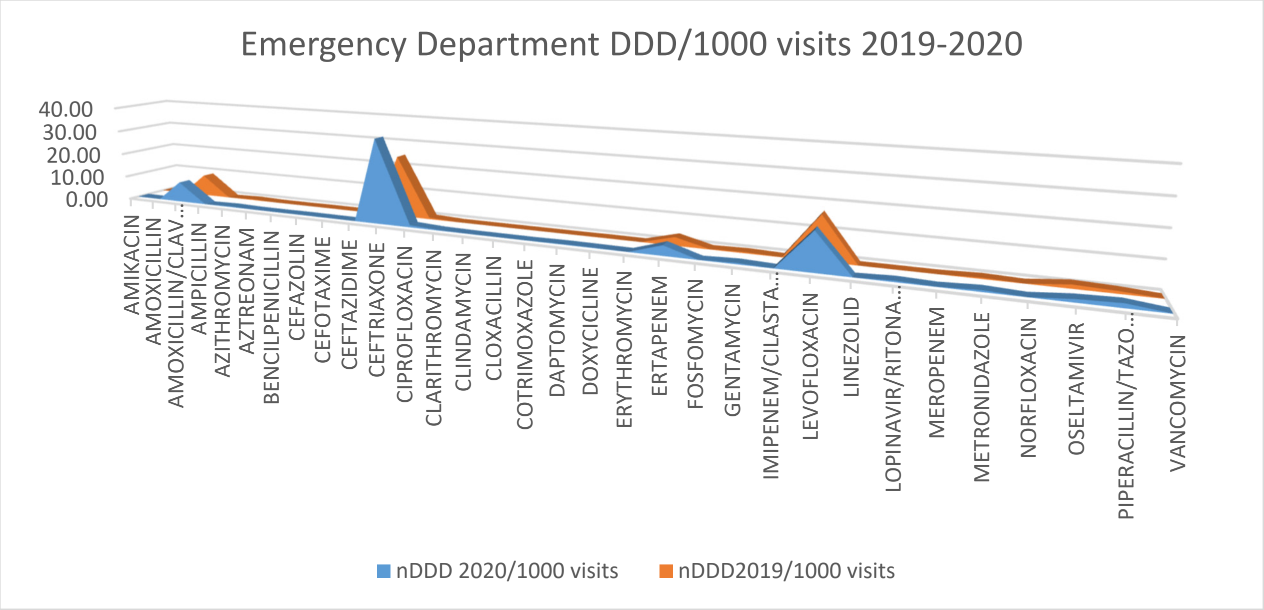

Figure S2. Antibiotic consumption: DDD/100 beds in internal medicine in 2019-2020

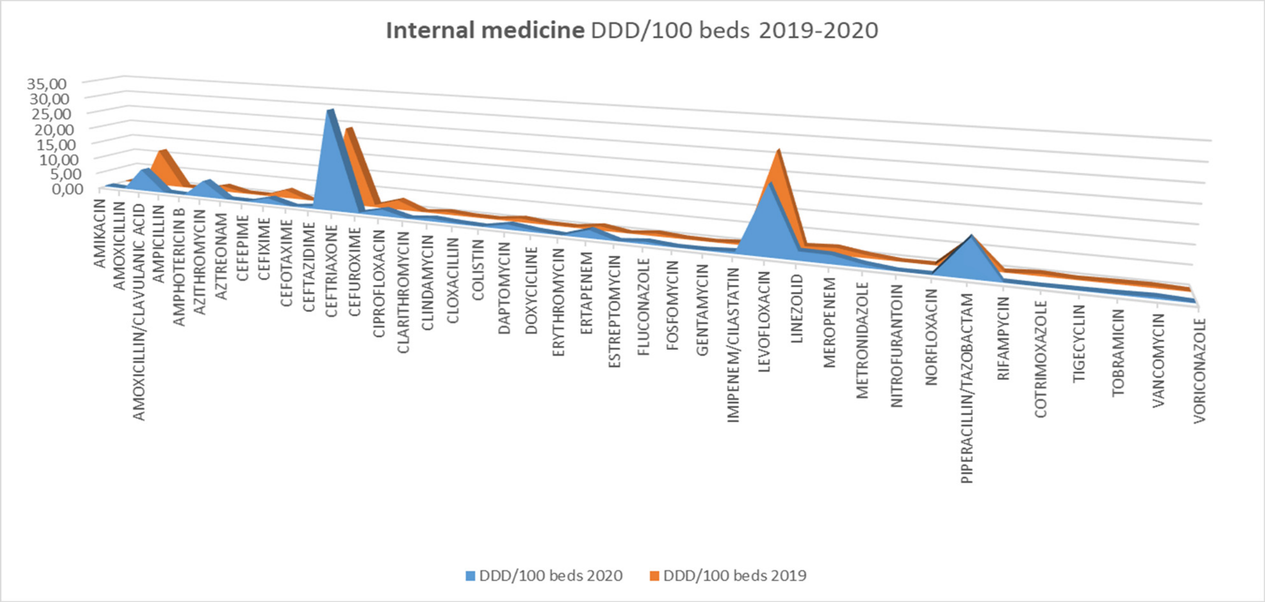

Figure S3. Antibiotic consumption: DDD/100 admissions in internal medicine in 2019-2020

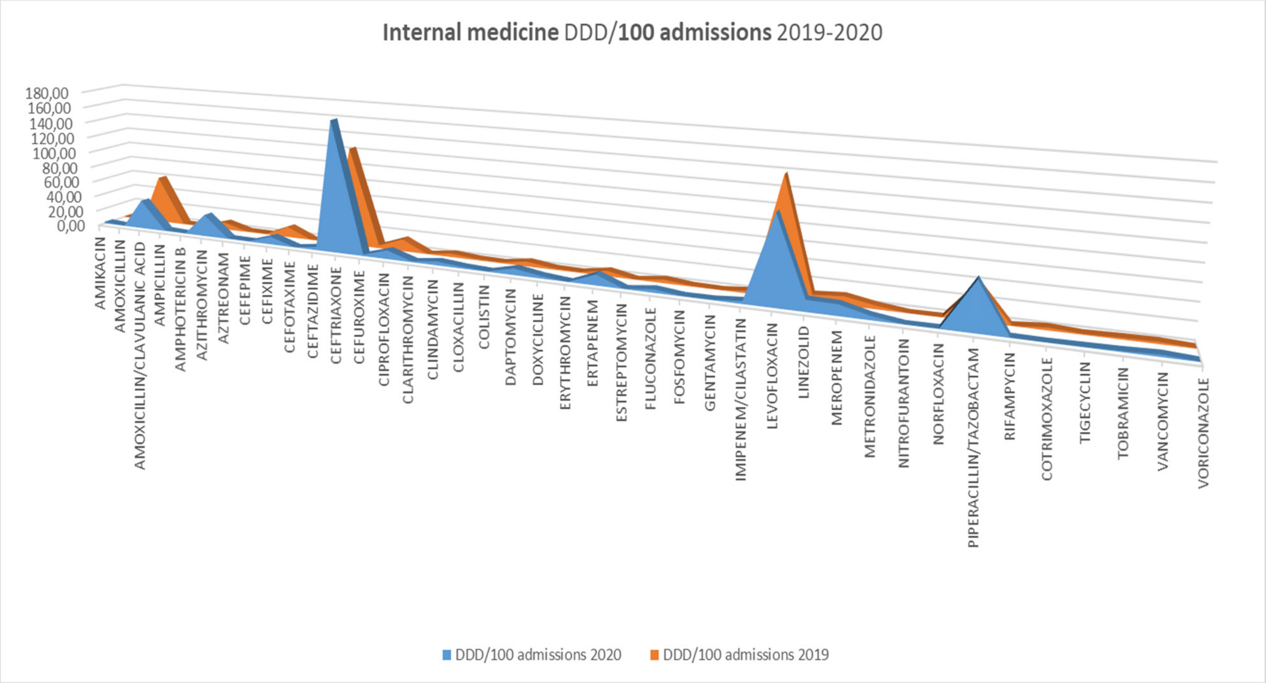

Table S1. Distribution of treatment duration

Treatment duration	Number of patients	Mean	Standard deviation	Confidence interval
**Optimal**	**25**	10.32	2.98	(9.09 - 11.55)
**Excessive**	**25**	13.52	2.95	(12.30 - 14.74)
**Short**	**0**	(NA)	NA	(NA – NA)
**Doubtful**	**5**	18.60	10.45	(5.62 - 31.58)

NA: not applied
